# A Misdiagnosis of Traumatic Hypersupination of the Distal Radioulnar Joint: A Case Report

**DOI:** 10.1155/2013/209675

**Published:** 2013-12-10

**Authors:** Vipin Asopa, Robert J. Douglas, Andrew D. Saies, James S. Church

**Affiliations:** ^1^Chelsea and Westminster Hospital, 369 Fulham Road, London, SW10 9NH, UK; ^2^Sportsmed SA, 32 Payneham Road, Stepney, Adelaide, SA 5069, Australia

## Abstract

Traumatic hypersupination injury of the distal radioulnar joint is a rare injury, and occurs when sufficient supination force is applied to the joint so as to tear the volar radioulnar ligament, resulting in separation of the triangular fibrocartilage complex, and subluxation of the tendon of extensor carpi ulnaris. This allows the distal ulna to rotate such that the ulnar styloid comes to lie adjacent to the ulna notch of the radius. Treatment of this injury requires manipulation of the joint, under anaesthesia or sedation. We describe a case where posttraumatic radiological investigation of a patient with an anatomical variation of the wrist when in supination resembled a traumatic hypersupination injury of the distal radioulnar joint. A review of the literature has revealed this to be the first reported case of this type.

## 1. Introduction

In the normal wrist, supination occurs with rotation of the concave distal radius about the convex ulna in an arc of up to 220 degrees [1]. The interosseous membrane and triangular fibrocartilage act as stabilisers while the dorsal and volar radioulnar ligaments tighten to provide rotational stability [2, 3]. Posterior-anterior (PA) radiography of the normal wrist will show the ulnar styloid projecting away from the distal radioulnar joint (DRUJ) with the groove for the tendon of extensor carpi ulnaris (ECU) radial to the ulnar styloid [4]. On the normal anterior-posterior (AP) view, the ulnar styloid usually overlies the central portion of the distal ulna [5]. Following trauma, this complex can become disrupted and give rise to a traumatic hypersupination injury of the DRUJ, and on a PA radiograph, the ulna styloid can be found to be lying within the ulna notch of the radius.

## 2. Case Report

**Figure 1:**
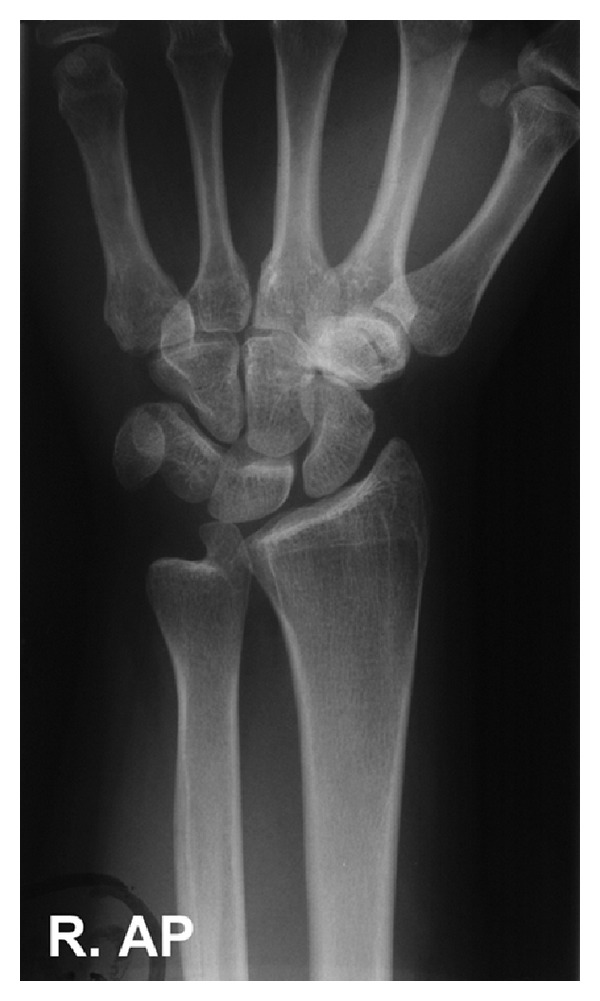
On arrival AP radiograph of the injured right wrist demonstrating the abnormal appearance of the ulnar styloid: it is lying adjacent to the ulnar notch of the radius.

A 19-year-old female was seen in the accident and emergency department following a fall onto her dominant right outstretched hand. There was no apparent hypersupination of the wrist at the time of injury. She reported pain in the wrist and denied previous trauma. On examination, the patient had mild swelling of the wrist with tenderness over the distal radioulnar joint, and limitation of forearm pronation. It was therefore not possible to obtain the standard PA wrist radiograph and an AP radiograph of the supine forearm was performed. This revealed the ulnar styloid to be lying adjacent to the ulnar notch of the radius (Figure 1). The clinical features and radiographic appearance suggested that the patient had suffered a traumatic DRUJ hypersupination injury.

**Figure 2:**
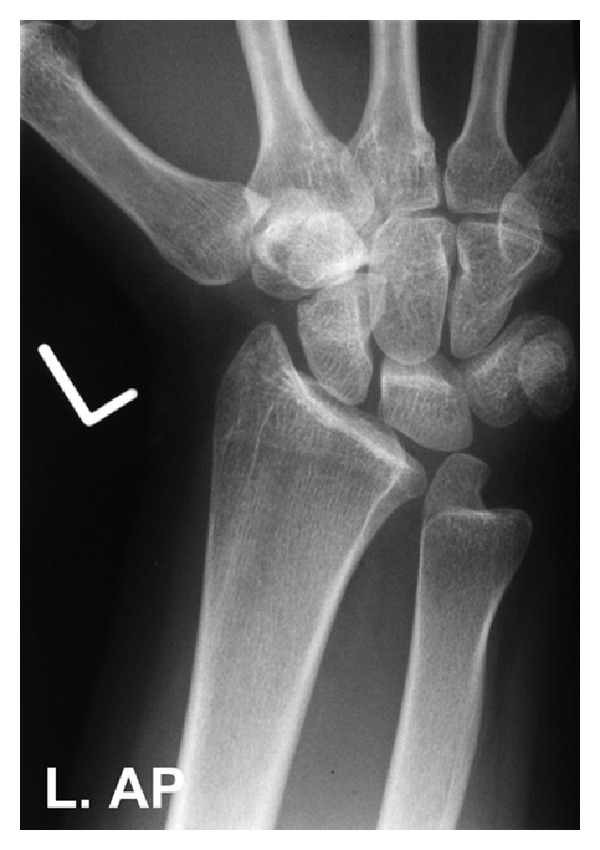
AP radiograph of the contralateral left wrist demonstrating the abnormal appearance of the ulnar styloid in supination. The lie of the ulnar styloid in supination is identical to that of the injured right wrist.

**Figure 3:**
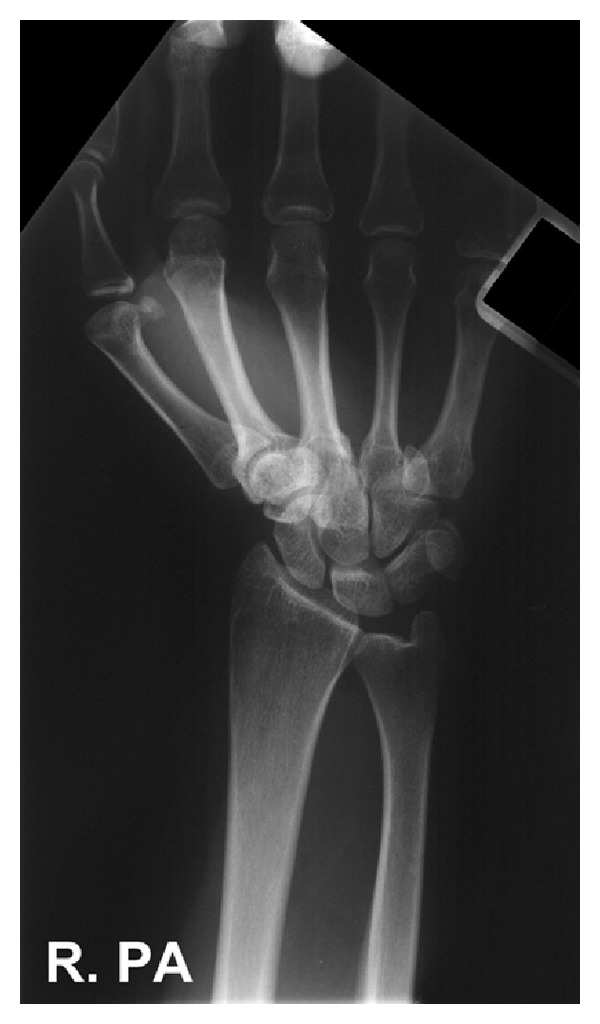
PA radiograph of the injured right wrist demonstrating normal anatomical alignment of the ulnar styloid.

The patient was admitted for a planned manipulation under anaesthesia. The following morning, she reported improvement of her wrist symptoms. The radiographs were reviewed by five experienced consultant orthopaedic surgeons who felt that the anatomical position of the ulnar styloid with the wrist in supination was abnormal. A computerised tomography (CT) scan was performed, which demonstrated no fracture. Radiographs of both wrists were then taken with the forearm in pronation and supination. An AP radiograph of the uninjured left wrist was similar in appearance to the right (Figure 2), while a PA (pronation) view of the injured wrist was normal (Figure 3).

## 3. Discussion

**Figure 4:**
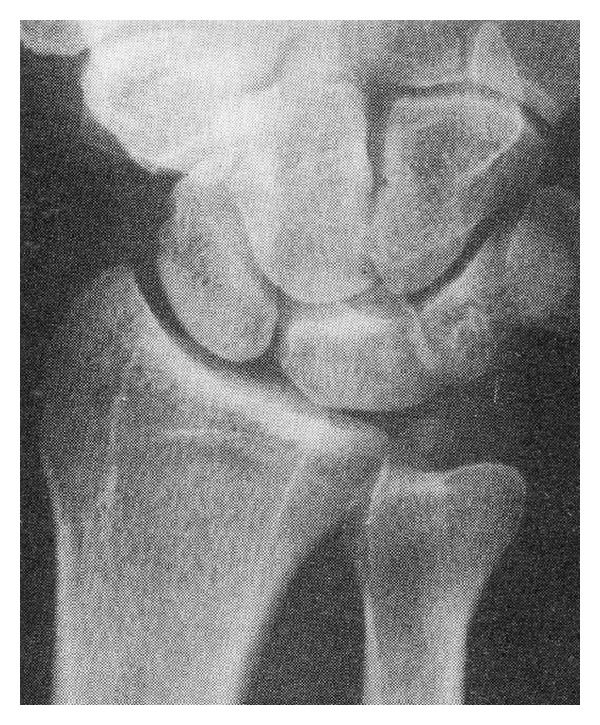
PA view of the right wrist demonstrating the abnormal lie of the ulnar styloid, following a hypersupination injury. (Reproduced by the kind permission of the British Editorial Society of Bone and Joint Surgery, from Graham et al. [2]).

In this case, PA X-ray of the right wrist undertaken in the ED demonstrated that the ulnar styloid was found to lie within the ulnar notch of the ulna. A review of the radiological literature [6] revealed that this has only previously been described in cases of traumatic hypersupination (Figure 4). Cadaveric studies have demonstrated that hypersupination injury of the DRUJ causes the tearing of the volar radioulnar ligament, separation of the triangular fibrocartilage complex, and subluxation of the tendon of ECU, allowing the radius to rotate such that the ulnar styloid lies within the sigmoid notch [2, 7].

## 4. Conclusion

Given that the patient made a rapid recovery, and the symmetrical appearance of the AP wrist radiographs, it must be concluded that this case represents an anatomical variation of normal. To the best of our knowledge, this appears to be the first reported case of an anatomical variation of this type.

Our “take home message” for the emergency physician, orthopaedic surgeon, and radiologist is to be aware of the possibility of variations of this type, and to radiographically examine the contralateral joint prior to pursuing other radiological modalities if there is any doubt in the initial diagnosis.
